# Choroidal vascularity index in type-2 diabetes analyzed by swept-source optical coherence tomography

**DOI:** 10.1038/s41598-017-18511-7

**Published:** 2018-01-08

**Authors:** Mirinae Kim, Min Ji Ha, Seung Yong Choi, Young-Hoon Park

**Affiliations:** 1 Department of Ophthalmology and Visual Science, College of Medicine, The Catholic University of Korea, Seoul, Republic of Korea; 2 Catholic Institute for Visual Science, College of Medicine, The Catholic University of Korea, Seoul, Korea

## Abstract

The relationships between changes in choroidal vasculature and the severity of diabetic retinopathy (DR) remain unclear. We assessed choroidal changes in diabetic patients by measuring choroidal vascularity index (CVI) in conjunction with DR stage. In this study, patients with diabetes and healthy controls were retrospectively analyzed. Subjects were divided into seven groups as follows: Healthy controls, no DR, mild/moderate non-proliferative DR (NPDR), severe NPDR, proliferative DR (PDR), panretinal photocoagulation-treated DR, and clinically significant macular edema. The mean CVI values in the above groups were 69.08, 67.07, 66.28, 66.20, 63.48, 65.38, and 66.28, respectively. The eyes of diabetic patients exhibited a significantly lower CVI value than those of healthy controls even without DR. The PDR group exhibited a significantly lower CVI value than the healthy control, no DR, and mild/moderate NPDR groups. Age, sex, disease duration, glycated hemoglobin, fasting blood sugar, or intraocular pressure had no correlation with CVI. In multivariate regression analysis, thicker subfoveal choroid and thinner central retina were significantly associated with higher CVI values. These findings carefully suggest that changes in choroidal vasculature could be the primary event in diabetes even where there is no DR.

## Introduction

The prevalence of type-2 diabetes mellitus (DM) has increased markedly in young adolescents worldwide. In 2010, the World Health Organization estimated that the global prevalence of DM is approximately 6.4%, equating to 280 million people^1^. It is estimated that the number of people with DM worldwide will double by 2025, resulting in 300 million with the condition^2^.

It is well known that DM is always accompanied by long-term complications, and diabetic retinopathy (DR), a microvascular complication, is one of the most frequent^3^. The prevalence of DR and vision-threatening DR have been reported as 28.5–33.2% and 4.4%, respectively^4,5^. The prevention and early detection of these complications are key issues, because DR is one of the leading causes of blindness in people aged 20–74 years in the US.

Currently, the gold-standard for DR grading is a modified version of the Early Treatment Diabetic Retinopathy Study (ETDRS) adaptation of the Modified Airlie House Classification^6^. Fluorescein angiography and indocyanine green angiography have been combined for evaluating retinal and choroidal vasculature^7^. In recent years, a quantum leap in ocular imaging technique has been achieved. Enhanced depth imaging of spectral-domain optical coherence tomography (OCT) facilitates the measurement of choroidal thickness (CT) and the characterization of changes in the choroidal vessels in several chorioretinal diseases^8^. Recent advances in swept-source (SS)-OCT systems utilizing longer wavelengths (usually approximately 1,050 nm) and faster scanning speeds have improved tissue penetration, spatial resolution, and signal-to-noise ratio^9^, allowing better visualization of the choroidal vasculature, even microvascular changes.

Several choroidal alterations in diabetic patients have been described in the literature^10–14^. Recently there has been a focus on CT, which differs significantly from that of healthy patients; however, inconsistent results have been reported. Several investigators have reported that CT was significantly reduced in mild or moderate non-proliferative DR (NPDR)^10,14^. Conversely, others reported that subfoveal CT (SFCT) increased as DR worsened to proliferative DR (PDR), and decreased in diabetic macular edema (DME) or laser-treated eyes^11,12^. Choroidal blood flow increased in NPDR patients, and decreased in laser-treated PDR patients^13^. These discrepancies are likely caused by the effect of various factors on CT^15–18^. Furthermore, it remains uncertain whether CT changes are influenced by remodeling of choroidal vasculature or altered stromal elements.

Recent studies have assessed the choroidal vascularity index (CVI), the ratio of the luminal area (LA) to the total choroidal area (TCA)^19–22^. CVI can indirectly measure choroidal vascularity quantitatively, overcoming the limitation of using CT alone. The relationships between changes in choroidal vasculature and the severity of DR are yet to be systematically investigated. It would be interesting to explore the vascular component via CVI, and evaluate CT in diabetic patients in conjunction with DR severity. Here, we evaluated CVI and CT according to DR stage, and aimed to determine whether choroidal vascular alterations in DM influence CT.

## Results

### Demographic and clinical characteristics

A total of 230 eyes were included in this study. Eyes were divided into seven groups as follows: Healthy controls (group 1, *n* = 45), no DR (group 2, *n* = 30), mild or moderate NPDR (group 3, *n* = 41), severe NPDR (group 4, *n* = 40), PDR (group 5, *n* = 8), panretinal photocoagulation (PRP)-treated DR (group 6, *n* = 35), and clinically significant macular edema (CSME) (group 7, *n* = 31). The demographic, ocular, and systemic characteristics of the subjects are shown in Table 1. Of the 185 type-2 diabetic patients whose eyes were included in the study, 105 were women and 80 were men, and their mean age was 58.04 ± 12.96 years. The mean duration of diabetes was 10.96 ± 7.42 years. The study groups did not differ significantly in terms of age or sex. Disease duration (*P* = 0.006), glycated hemoglobin (HbA1c) level (*P* < 0.001), and fasting blood glucose level (*P* = 0.043) did differ significantly between each DR group (Kruskal Wallis test). BCVA (logMAR) was significantly lower in the PRP-treated DR (*P* < 0.001) and CSME (*P* < 0.001) groups. Intraocular pressure (IOP) was significantly higher in the PDR (*P* = 0.047) and PRP-treated DR groups (*P* = 0.001).

**Table 1:** Demographic and clinical characteristics of the eyes (n = 230), organized by study group

Variables	Healthy controls (n = 45)	No DR (n = 30)	Mild or moderate NPDR (n = 41)	Severe NPDR (n = 40)	PDR (n = 8)	PRP treated DR(n = 35)	CSME(n = 31)	*P* value
No. of eyes	45	30	41	40	8	35	31	—
Age, years	57.47 ± 12.87	57.50 ± 15.65	59.17 ± 13.30	59.83 ± 12.26	58.50 ± 6.78	54.09 ± 12.77	59.10 ± 10.62	0.058
Sex, n (%)								0.232
Female	33	16	21	23	3	24	18	
Male	12	14	20	17	5	11	13	
Years with diabetes	—	6.97 ± 6.89	12.59 ± 9.44	11.56 ± 5.51	12.25 ± 5.63	12.57 ± 7.21	9.74 ± 6.44	**0**.**006**
HbA1c,%	—	6.60 ± 0.86	7.43 ± 1.27	7.60 ± 1.31	7.73 ± 0.36	8.34 ± 2.02	7.11 ± 1.19	**<0**.**001**
Fasting blood sugar	—	138.79 ± 50.78	150.31 ± 49.65	167.94 ± 53.22	153.38 ± 38.10	174.66 ± 49.52	153.00 ± 63.07	**0**.**043**
Systolic BP	—	123.39 ± 12.86	129.17 ± 13.33	126.00 ± 12.90	128.00 ± 8.91	124.52 ± 9.65	121.67 ± 16.04	0.243
Diastolic BP	—	77.71 ± 9.93	77.69 ± 7.55	74.81 ± 12.39	81.63 ± 9.33	79.28 ± 9.76	72.63 ± 11.71	0.119
Ophthalmologic examination								
BCVA, logMAR	0.07 ± 0.11	0.06 ± 0.09	0.07 ± 0.10	0.09 ± 0.11	0.08 ± 0.09	0.22 ± 0.18	0.34 ± 0.31	**<0**.**001**
SE, diopter	−0.71 ± 1.88	−0.31 ± 1.61	−0.11 ± 1.52	−0.59 ± 1.51	−0.27 ± 2.85	−1.15 ± 1.96	−0.44 ± 1.53	0.157
IOP, mmHg	14.51 ± 2.99	15.27 ± 3.36	14.34 ± 3.23	15.13 ± 2.89	17.00 ± 3.12	17.17 ± 3.34	14.81 ± 2.94	**0**.**005**

Data are expressed as mean ± standard deviation (95% confidence interval).

Statistically significant *P* values are highlighted as bold (Kruskal Wallis test).

BCVA, best-corrected visual acuity; BP, blood pressure; CSME, clinically significant macular edema; DR, diabetic retinopathy; IOP, intraocular pressure; logMAR, logarithm of the minimum angle of resolution; NPDR, non-proliferative diabetic retinopathy; PDR, proliferative retinopathy; PRP, panretinal photocoagulation; SE, spherical equivalent.

### CT and CVI

**Figure 1:**
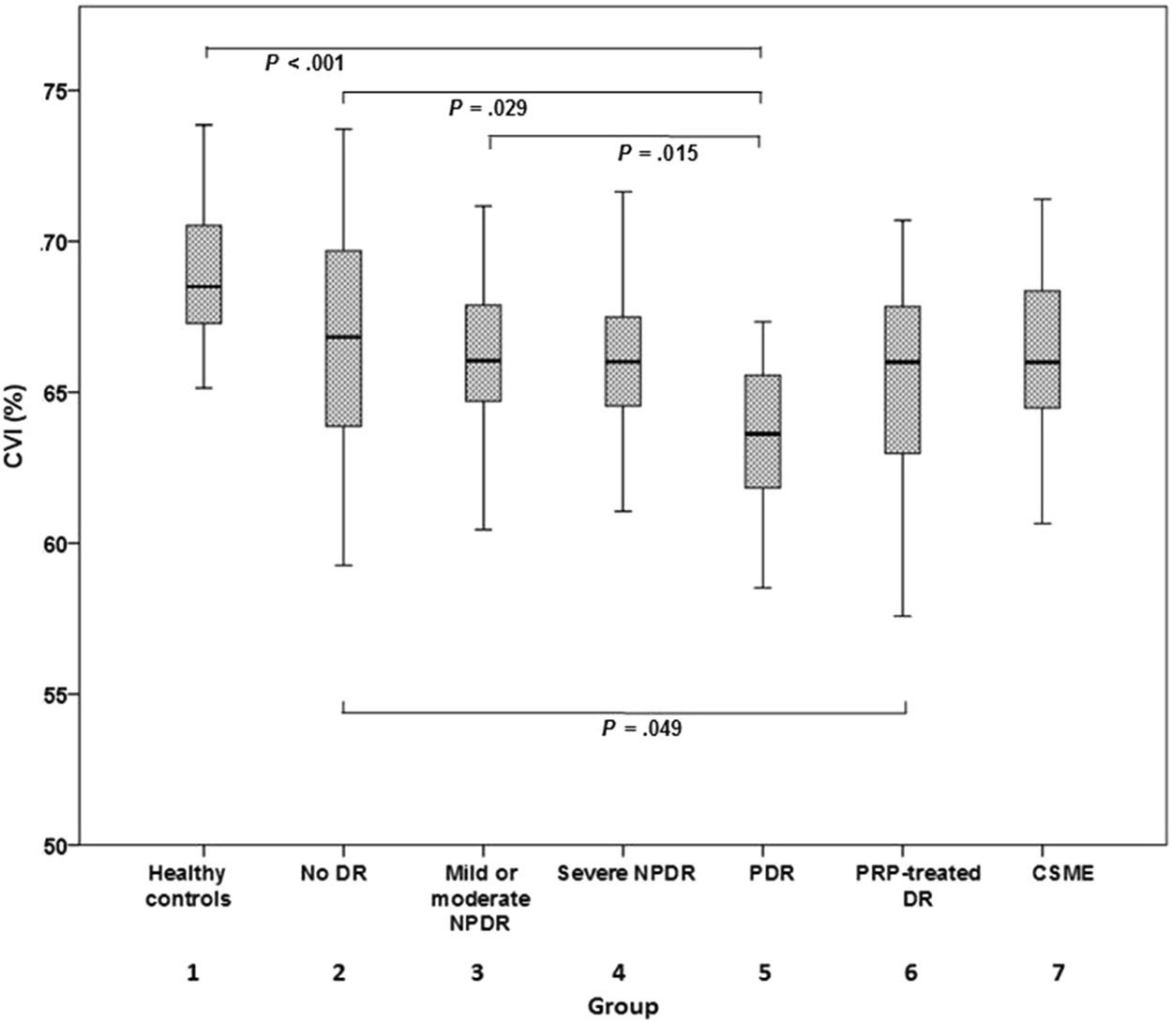
Choroidal vascularity index in each group. The CVIs in groups 1–7 were 69.08 ± 2.29, 67.07 ± 3.71, 66.28 ± 2.70, 66.20 ± 2.56, 63.48 ± 2.89, 65.38 ± 3.15, and 66.28 ± 2.85, respectively. Relative to the eyes of healthy controls, the eyes of diabetic patients exhibited a significantly lower mean CVI (*P* < 0.001). Notably, the PDR group exhibited a significantly lower mean CVI relative to groups 1 (*P* < 0.001), 2 (*P* = 0.029), and 3 (*P* = 0.015). Group 6 exhibited a significantly lower mean CVI than group 2 (*P* = 0.049), but it did not differ significantly from that of any of the other DR groups. No significant change in CVI was observed in the CSME group in comparison with the other DR groups. Note: CSME, clinically significant macular edema; CVI, choroidal vascularity index; DR, diabetic retinopathy; NPDR, non-proliferative diabetic retinopathy; PDR, proliferative diabetic retinopathy; PRP, panretinal photocoagulation.

With regard to choroidal characteristics, Fig. 1 shows the CVI measurements of each group. The CVIs in groups 1–7 were 69.08 ± 2.29, 67.07 ± 3.71, 66.28 ± 2.70, 66.20 ± 2.56, 63.48 ± 2.89, 65.38 ± 3.15, and 66.28 ± 2.85, respectively. Relative to the eyes of healthy controls, the eyes of diabetic patients exhibited a significantly lower mean CVI (*P* < 0.001). Notably, the PDR group exhibited a significantly lower mean CVI relative to groups 1 (*P* < 0.001), 2 (*P* = 0.029), and 3 (*P* = 0.015). Group 6 exhibited a significantly lower mean CVI than group 2 (*P* = 0.049), but it did not differ significantly from that of any of the other DR groups. No significant change in CVI was observed in the CSME group in comparison with the other DR groups.

**Figure 2:**
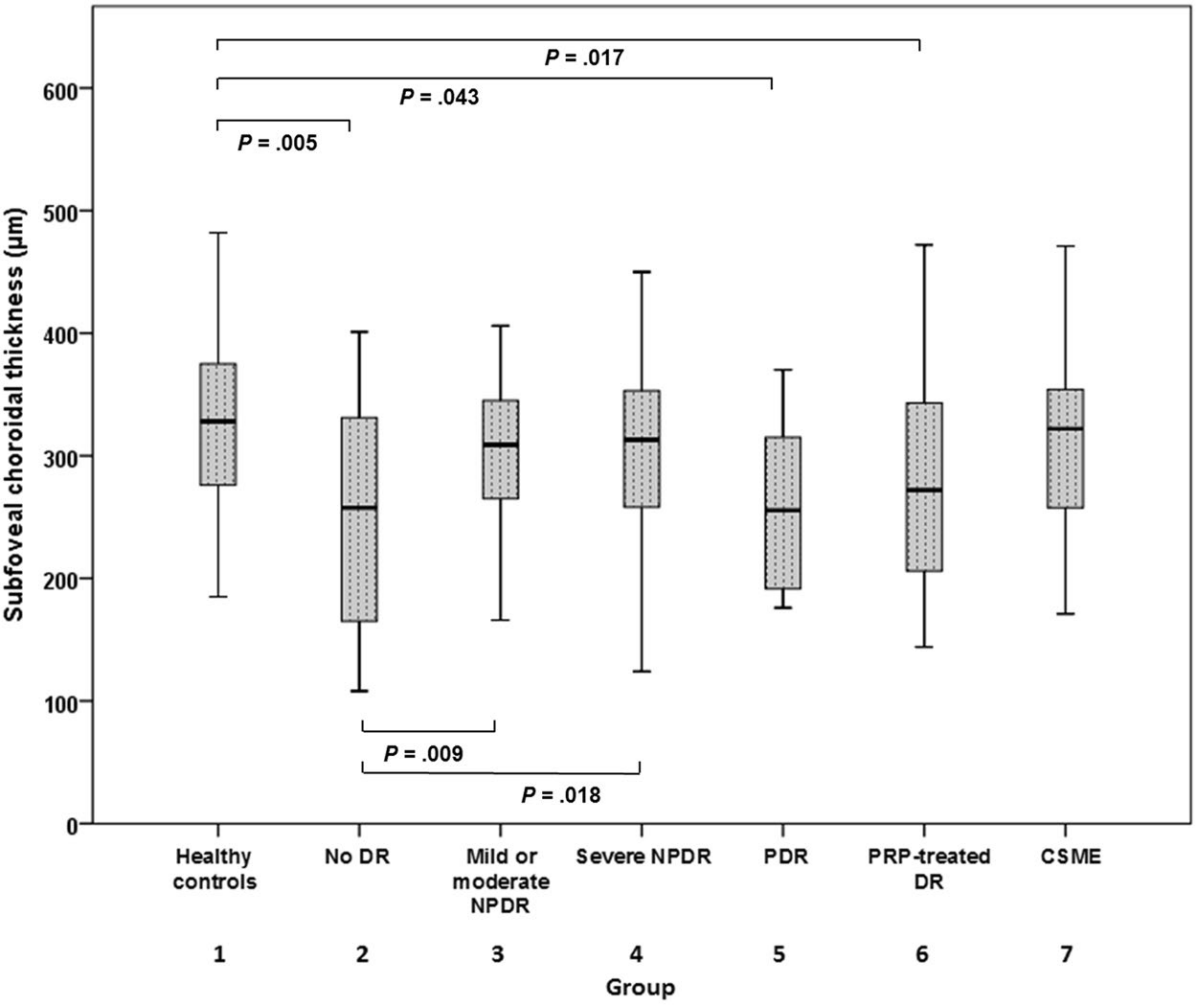
Subfoveal choroidal thickness in each group. The mean subfoveal choroidal thicknesses in groups 1–7 were 320.00 ± 77.92, 258.13 ± 89.02, 310.22 ± 72.41, 304.53 ± 69.26, 258.75 ± 73.29, 276.29 ± 79.51, and 312.58 ± 89.59, respectively. A crude analysis of these data suggested that eyes of diabetic patients exhibited a lower CT than eyes of healthy controls. Among the eyes of diabetic patients, the lowest CT values were observed in the PDR group. The statistical significances are shown in the figure. Note: CSME, clinically significant macular edema; CT, choroidal thickness; DR, diabetic retinopathy; NPDR, non-proliferative diabetic retinopathy; PDR, proliferative diabetic retinopathy; PRP, panretinal photocoagulation.

We determined retinal thickness (RT) and CT in each ETDRS grid sector (Supplementary Table). Figure 2 shows the subfoveal CT measurements of each group. A crude analysis of these data suggested that eyes of diabetic patients exhibited a lower CT than eyes of healthy controls. Among the eyes of diabetic patients, the lowest CT values were observed in the PDR group.

In univariate regression analysis, there were no significant correlations of age, sex, disease duration, HbA1c, fasting blood sugar, blood pressure (BP), or IOP with CVI. In the multivariate linear regression model, thicker subfoveal choroid (*P* = 0.003) and thinner central retina (*P* = 0.024) were significantly associated with higher CVI (Table 2).

**Table 2:** Linear regression analyses of factors associated with choroidal vascularity index.

Variables	Univariate		Multivariate^a^	
	Standardized β	*P* value	Standardized β	*P* value
Age, years	−0.075	0.255	—	—
Sex, female	−0.041	0.538	—	—
Years with diabetes	−0.059	0.424	—	—
HbA1c,%	−0.011	0.892	—	—
Fasting blood sugar	−0.094	0.244	—	—
Systolic BP	0.035	0.669	—	—
Diastolic BP	0.014	0.858	—	—
BCVA, logMAR	−0.144	0.029	−0.021	0.756
SE, diopter	0.127	0.054	0.098	0.112
IOP, mmHg	−0.109	0.101	—	—
Central RT, μm	−0.141	0.033	−0.151	**0**.**024**
Mean RT, μm	−0.014	0.837	—	—
Central CT, μm	0.476	<0.001	0.197	0.313
Mean CT, μm	0.458	<0.001	−0.029	0.881
Subfoveal CT, μm	0.487	<0.001	0.357	**0**.**003**

^a^Adjusted for variables with a *P* value < 0.10 in the univariate analysis. β, regression coefficient.

Statistically significant *P* values in multivariate regression analyses are highlighted as bold.

BCVA, best-corrected visual acuity; BP, blood pressure; CT, choroidal thickness; IOP, intraocular pressure; logMAR, logarithm of the minimum angle of resolution; RT, retinal thickness; SE, spherical equivalent.

### Effect of PRP on choroidal vascularity

To evaluate the effects of laser treatment on choroidal vascularity, we investigated eyes of 35 PRP-treated DR patients, including 15 severe NPDR patients and 20 PDR patients, separately. The mean CVI of the PRP-treated group did not differ significantly from that of the severe NPDR group (*P* = 0.340) or the PDR group (*P* = 0.283). There was no significant difference in CVI between the PRP-treated group and the DR group (*P* = 0.015) or the mild/moderate NPDR group (*P* = 0.144).

## Discussion

In the current study, we determined choroidal vascular changes in diabetic patients by measuring a quantitative parameter, “CVI”. A reduction in the choroidal vascular component was evident in type-2 DM patients, regardless of DR stage. Notably, the PDR group exhibited lower mean CVI values than those of the healthy control, no DR, and mild/moderate NPDR groups.

Tan *et al.* ^20^. evaluated CVI in DM patients, and their eyes exhibited reduced CVI values compared to controls (65.10 ± 0.20 versus 67.20 ± 0.16, *P* < 0.0001). However, they only included 38 eyes of diabetic patients and they did not analyze CVI according to DR stage. In our study population, the CVI was 66.10 ± 3.03 in the collective total of the eyes of diabetic patients, and 69.08 ± 2.29 in healthy controls. Figures 1 and 2 show the CVI and CT trend associated with disease progression. CVI decreased as DR progressed to PDR. In the early stages of DR, SFCT and CVI tended to diverge. In the mild/moderate NPDR group, SFCT was greater and CVI was significantly lower compared with the no DR group. Choroidal blood flow deficit can be an early pathologic change in DR, as shown in an animal model^23^. Furthermore, the size and density of choroidal vessels reduces with diabetic progression^24^, and choroidal blood flow reduces in diabetic patients before retinopathy manifestation^25^. These observations suggest that the thickening of the choroid in the early stages of DR is stromal thickening, and that vascular components do not contribute to it. As disease progressed to severe NPDR or PDR, SFCT and CVI tended to decrease.

The choroidal thinning associated with DR progression might be due to choriocapillary loss or vascular constriction secondary to choroidal hypoxia. It is unknown whether choroidal changes are the cause or result of retinopathy progression. As CVI was significantly lower even in the patients without DR, we hypothesized that ischemic changes in choroidal vasculature is the primary event in diabetes, even when DR is absent. Choroidal abnormalities in diabetic eyes include microaneurysms, choriocapillaris obstruction, vascular remodeling with increased tortuosity, vascular dropout, and areas of vascular non-perfusion^26^. Increased CT in early DR might be due to stromal thickening associated with these changes and the subsequent hyperpermeability of the choroidal vasculature. Thereafter, as disease progresses, persistent choroidal hypoxia may result in a decrease in CVI and CT.

To evaluate the effects of laser treatment on choroidal vascularity, we investigated eyes with a history of PRP separately. It has previously been reported that choroidal blood flow decreased markedly after PRP, possibly due to downregulation of vascular endothelial growth factor^27^. Some investigators have reported reductions in CT in the eyes of DME patients^28^, whereas others have reported increases^12^. We found no significant difference in CVI between eyes with and without DME.

Choroidal changes in diabetic patients might play an important role in the development of DR, because the choroid is responsible for supplying blood to the outer retinal layer^10^. Several choroidal changes have been described in diabetic patients, but reports on CT in such eyes are discrepant. The analysis of choroidal changes using CT may have some limitations, because various factors, such as age, sex, refractive error, systolic BP, axial length, anterior chamber depth, and lens thickness may affect CT^15,17^. Furthermore, diurnal variations of approximately 20–30 µm in CT have been reported^16,18^. The analysis of choroidal changes using CT may have low reproducibility and low reliability. In those respects, CVI may be a more stable and objective quantitative marker for the assessment of choroidal vascularity, which may overcome the limitations associated with using CT alone. There is no concrete evidence that the low reflective dark areas represent the vascular lumens and the light areas represent the stromal areas. But the findings of previous reports and that of empirical observations suggest that the low reflective dark areas were the vascular components in the binarized images^29,30^.

Our study had some limitations. First, we measured only the central 1,500 μm of the scan as a representative area. A larger area may have provided more representative information on the CVI. Second, we measured only a single scan going through the fovea, as a single foveal scan-based CVI represents the total macular CVI in healthy population^31^. A 3D volume scan over the macular area may have provided more robust information relating to the disease profile. Third, some authors have reported significant diurnal variation in CT measurements obtained via OCT. The circadian variation of choroidal vascularity is unknown, but this might have affected our results. And we did not incorporate the axial length. Previous studies showed that CVI is not significantly affected by axial length, but it might be related to the choroidal thickness. Fourth, we only included 8 PDR patients. This was because we only included treatment-naive PDR patients and we excluded cases of PDR with DME or media opacity such as vitreous haemorrhage. These limitations should be addressed in future studies.

In conclusion, CVI can be used as a quantitative parameter for choroidal evaluation. The eyes of patients with diabetes, even without DR, exhibited a significantly lower CVI than those of healthy controls. Notably, the PDR group exhibited a significantly lower mean CVI relative to the other DR stages. Future longitudinal data collection and correlation with fluorescein angiography or indocyanine green angiography are necessary to corroborate the results of the current study.

## Methods

### Study population

This retrospective observational study adhered to the tenets of the Declaration of Helsinki, and all protocols were approved by the institutional review board of the Catholic University of Korea. The requirement for obtaining informed patient consent was waived due to the retrospective nature of the study.

The study included 185 eyes of patients with a confirmed diagnosis of type-2 DM, and 45 eyes of healthy controls. All participants were recruited between December 2016 and April 2017 at Seoul St. Mary’s Hospital in Korea, and a retrospective chart review was conducted. Exclusion criteria were as follows: (1) Refractive errors of more than ± 6 diopters (as spherical equivalent), (2) eyes with a history of any ocular trauma, laser treatment, or intraocular surgery, (3) eyes with a history of intravitreal or sub-Tenon’s injections, (4) other systemic disease that could affect the eye, (5) presence of other retinal diseases, including glaucoma, age-related macular degeneration, retinal vein occlusion, or neurodegenerative disease, (6) media opacity that could affect image quality, (7) pachychoroidal pigment epitheliopathy in the fellow eye (such as central serous chorioretinopathy or polypoidal choroidal vasculopathy)^32^, and (8) any history of uveitis.

To evaluate the effect of PRP on CT, eyes with a history of PRP were considered separately. However, patients treated within the past 1 year were excluded to avoid possible error due to choroidal swelling induced by laser treatment. Healthy subjects were recruited from consecutive patients scheduled for routine ocular examination for refractive error correction at Seoul St. Mary’s Hospital in Korea.

Demographic information and comprehensive medical and ophthalmologic history were recorded at the initial visit. All subjects underwent ocular examinations, including best-corrected visual acuity (BCVA) evaluation (logarithm of the minimum angle of resolution scale, logMAR), non-contact pneumatic tonometry, slit-lamp biomicroscopy, dilated fundus examination, and OCT. Imaging was performed with a SS-OCT device (DRI Triton, Topcon, Tokyo, Japan) using a 1050-nm wavelength light source, and a scanning speed of 100,000 A-scans/second. A 6-line radial pattern scan (1024 A-scans) centered on the fovea was obtained from each eye.

### DR grading

The DR grade was classified as no DR, mild/moderate NPDR, severe NPDR, or PDR, according to the modified ETDRS retinopathy severity scale^6^. Eyes that had been treated with PRP for at least 1 year before the study were allocated to the PRP-treated DR group. CSME is defined as retinal thickening that involves or threatens the center of the macula (even if visual acuity is not yet reduced), and is assessed via stereoscopic biomicroscopy or stereoscopic photography according to the criteria described by the ETDRS, and confirmed via SS-OCT^33^. Cases of DME with apparent epiretinal membrane or vitreo-macular traction were excluded.

### OCT image acquisition and CVI assessment

Retinal thicknesses and CTs were obtained with the automatic built-in software associated with the SS-OCT device. Thickness maps were created in accordance with the conventional ETDRS grid with 9 independent sectors. The choroid was segmented in the B-scans, after identification of the outer border of the retinal pigment epithelium band and the choroid−scleral junction as the inner and outer boundaries of the choroid, respectively.

SFCT was manually measured at the foveal center using digital calipers provided by the OCT software. SFCT was measured by calculating the distance from a hyper-reflective line representing the outer border of the retinal pigment epithelium to the inner edge of the suprachoroidal space, which was represented by a hypo-reflective line on OCT images^34^. To avoid interobserver variation, two experienced independent observers measured SFCT and the average value was used for analysis.

**Figure 3:**
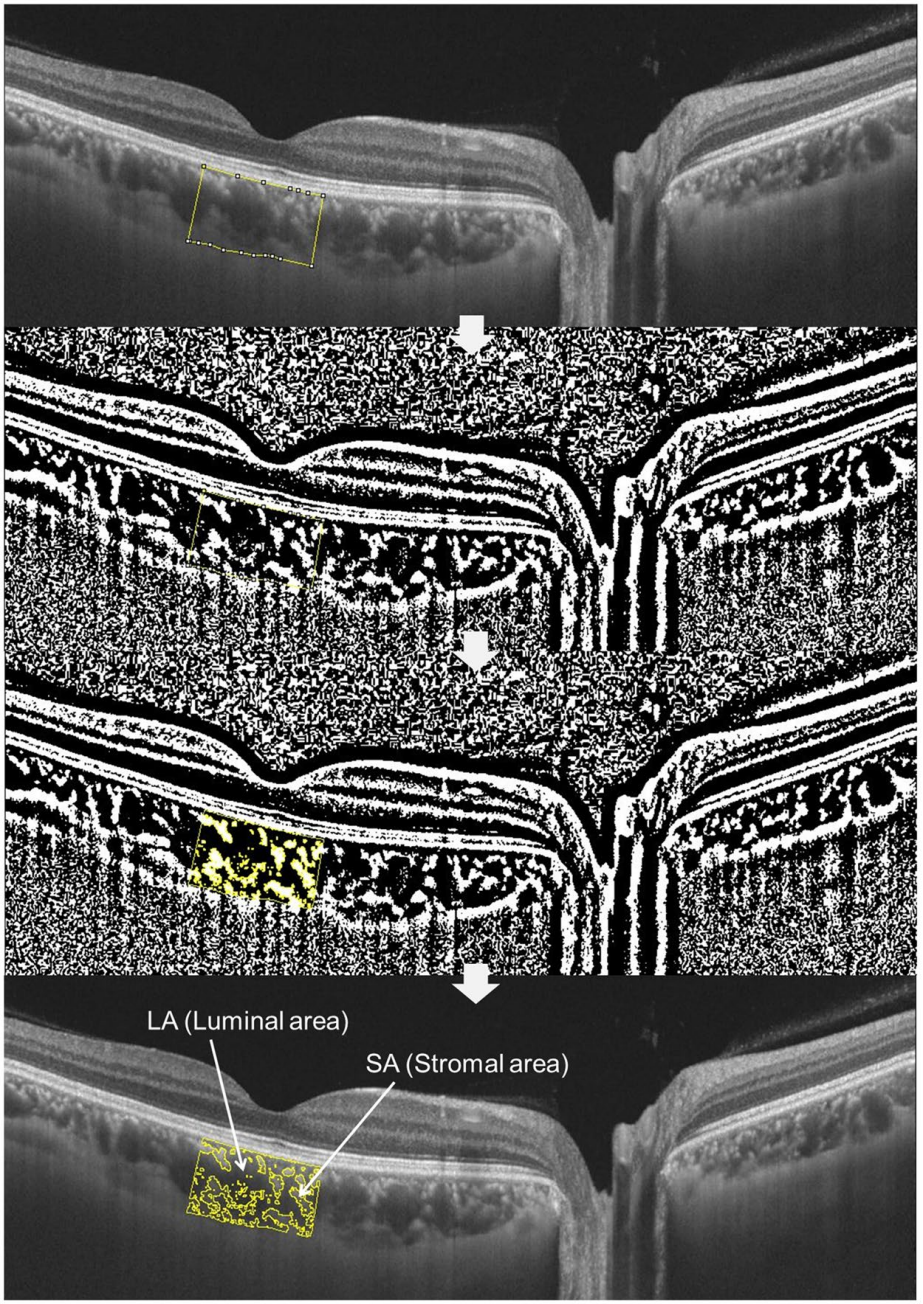
Representative image processing to obtain choroidal vascularity index. Using the polygon selection tool, the total choroidal area (TCA) for the subfoveal region within a width of 1500 μm (750 μm either side of the fovea) was selected, and regions of interest (ROIs) were added to the ROI manager. After converting the image to an 8-bit image, a Niblack autolocal threshold tool was applied, which yielded the mean pixel value with the standard deviation (SD) for all the points. The luminal area (LA) was highlighted by applying the color threshold, and subsequently added to the ROI manager. To determine the LA within the initially selected polygon, both the areas in the ROI manager were selected and merged via an “AND” operation. This composite third area was added to the ROI manager. The first area represented the total of the choroid selected or TCA, and the third composite area represented the vascular area or LA. The ratio of the LA to the TCA was termed the CVI. (LA + SA = TCA).

To assess CVI, the raster scan passing through the fovea was selected for image binarization. It was segmented using the protocol described by Agrawal *et al*.^19^, and image binarization was performed using Image J software (Version 1.51; https://imagej.nih.gov/ij/). A representative depiction of image processing to obtain CVI is shown below in Fig. 3. Using the polygon selection tool, the total choroidal area (TCA) for the subfoveal region within a width of 1500 μm (750 μm either side of the fovea) was selected, and regions of interest (ROIs) were added to the ROI manager. After converting the image into 8 bit, a Niblack autolocal threshold tool^35^ was applied, which gives the mean pixel value with the standard deviation (SD) for all the points. LA was highlighted by applying the color threshold, and subsequently added to the ROI manager. To determine the LA within the initially selected polygon, both the areas in the ROI manager were selected and merged via an “AND” operation. This composite third area was added to the ROI manager. The first area represented the total of the choroid selected or TCA, and the third composite area represented the vascular area or LA. The ratio of LA to TCA was deemed to be the CVI.

### Statistical analysis

An exploratory analysis was conducted for all variables. Categorical data are expressed as absolute numbers, and continuous data as mean ± SD (95% confidence interval). Statistical analysis was performed using the Statistical Package for the Social Sciences for Windows ver. 23.0 (SPSS Inc., Chicago, IL, USA). The normality of data distribution was confirmed via the Kolmogorov–Smirnov test. Student’s *t*-test was used to compare variables between groups, and the Kruskal−Wallis, nonparametric chi-square, and Mann−Whitney tests were used as appropriate. Univariate and multiple linear regression analyses were performed to assess associations between the CVI (dependent variables) and ocular and systemic factors (independent variables). For multiple linear regression, factors showing significant associations in univariate analysis (*P* < 0.10) were included. All *P-*values were 2-sided, and *P*-values < 0.05 were considered significant.

### Availability of data and materials

The datasets during and/or analyzed during the current study are available from the corresponding author on reasonable request.

## Electronic supplementary material

Supplementary Dataset
