# Supplementary material for: Choroidal vascularity index in type-2 diabetes analyzed by swept-source optical coherence tomography
Source: Sci Rep. 2018 Jan 8;8:70. doi: 10.1038/s41598-017-18511-7 (PMC5758605; doi:10.1038/s41598-017-18511-7)
Supplement: Supplementary file 1 — Supplementary Dataset [file 41598_2017_18511_MOESM1_ESM.pdf]

Choroidal vascularity index in type-2 diabetes analyzed by swept-source optical coherence tomography

Mirinae Kim,[1] Min Ji Ha,[1] Seung Yong Choi,[1] Young-Hoon Park[1,2*]

[1]Department of Ophthalmology and Visual Science, College of Medicine, The Catholic University of Korea, Seoul, Republic of Korea (M.K., M.J.H., S.Y.C., Y.H.P.);

[2]Catholic Institute for Visual Science, College of Medicine, The Catholic University of Korea, Seoul, Korea (Y.H.P)

Supplementary Table. The retinal and choroidal thicknesses, and choroidal vascularity index (CVI) by group

Variables	Healthy controls (n = 45)	No DR (n = 30)	Mild or moderate NPDR (n = 41)	Severe NPDR (n = 40)	PDR (n = 8)	PRP treated DR (n = 35)	CSME (n = 31)
CVI (%)	69.08±2.29	67.07±3.71	66.28±2.70	66.20±2.56	63.48±2.89	65.38±3.15	66.28±2.85
Retinal thickness (ETDRS sector)							
Central	221.29±20.42	230.40±15.17	236.29±23.68	237.83±22.08	237.13±35.93	245.86±39.58	307.48±81.74
Inner superior	311.89±16.84	309.03±15.95	311.46±15.40	318.75±27.89	317.25±26.46	309.87±25.42	354.77±60.19
Inner nasal	311.31±19.36	309.23±15.50	311.61±17.24	312.80±24.21	315.13±30.94	312.00±29.66	351.19±58.12
Inner inferior	309.29±16.61	306.53±14.89	308.90±16.10	311.03±23.99	308.25±32.48	310.09±28.38	344.55±36.43
Inner temporal	299.87±14.68	299.03±15.83	303.73±14.39	306.35±21.69	302.25±27.43	307.11±31.69	357.77±59.69
Outer superior	277.40±12.97	270.57±14.55	275.73±15.11	290.15±37.59	296.88±38.26	284.14±3.79	324.13±42.88
Outer nasal	292.71±18.06	287.07±13.96	286.83±13.90	295.03±31.41	304.25±23.25	302.09±28.96	326.06±44.57

Outer inferior	263.84±12.13	255.67±13.61	258.93±13.61	271.35±28.51	267.38±22.64	274.34±25.29	296.10±30.89
Outer temporal	262.76±11.36	260.00±25.98	264.02±17.72	276.38±37.32	270.63±23.81	280.29±31.85	326.39±62.53
Mean all sectors	283.37±13.59	280.84±13.80	284.17±12.88	291.07±24.16	291.01±25.79	291.76±23.42	332.05±37.15
Choroidal thickness (ETDRS sector)							
Central	267.27±87.42	216.00±90.11	241.03±77.52	241.11±81.14	202.13±60.92	229.68±78.85	252.84±88.63
Inner superior	255.27±78.19	208.88±82.33	242.03±70.25	233.60±78.23	201.75±54.94	226.13±80.81	260.29±89.70
Inner nasal	250.06±93.99	201.00±89.66	222.63±82.91	238.22±81.42	181.25±65.87	223.36±88.87	244.90±91.23
Inner inferior	274.27±88.96	211.21±86.13	229.29±90.28	250.51±85.24	197.50±63.88	224.94±81.35	248.32±92.44
Inner temporal	264.77±76.22	209.88±78.26	243.14±73.00	232.03±72.84	204.50±53.33	218.61±69.96	245.87±79.59
Outer superior	237.41±83.36	198.88±70.27	226.09±83.64	214.65±69.19	211.63±52.77	212.84±79.49	239.61±73.89
Outer nasal	195.53±91.59	162.63±82.28	174.34±78.65	193.05±73.72	141.63±63.45	183.55±87.69	205.74±82.73
Outer inferior	248.62±89.90	196.38±77.56	218.17±80.96	233.00±83.82	193.25±54.13	205.13±77.48	221.97±89.06
Outer temporal	239.32±67.16	197.58±66.61	222.03±65.07	211.87±70.33	203.25±38.89	191.74±63.64	223.07±85.36
Mean all sectors	248.06±77.76	200.27±76.89	224.31±73.33	227.56±70.22	192.99±52.62	212.86±73.35	238.07±78.73
Subfoveal choroidal thickness	320.00±77.92	258.13±89.02	310.22±72.41	304.53±69.26	258.75±73.29	276.29±79.51	312.58±89.59

Data are expressed as mean ± standard deviation (95% confidence interval).

CSME, clinically significant macular edema; CVI, chroidal vascularity index; DR, diabetic retinopathy; NPDR, non-proliferative diabetic retinopathy; PDR, proliferative retinopathy; PRP, panretinal photocoagulation.
